# Rapid and Simple RPHPLC Method for the Estimation of Metformin in Rat Plasma

**DOI:** 10.4103/0250-474X.41455

**Published:** 2008

**Authors:** M. M. Wanjari, A. W. There, M. R. Tajne, C. T. Chopde, S. N. Umathe

**Affiliations:** Department of Pharmaceutical Sciences, Rashtrasant Tukadoji Maharaj Nagpur University, Mahatma Jyotiba Fuley Shaikshanik Parisar, Amravati Road, Nagpur-440 033, India

**Keywords:** Acetonitrile, pharmacokinetics, diabetes, metformin

## Abstract

A simple reverse phase high-performance liquid chromatographic method has been developed for determining the concentration of metformin in rat plasma. The method employs C_18_ column (300 mm × 2.4 mm i.d.), ammonium acetate (0.15 M) and acetonitrile (90:10; pH-5.5; 1.0 ml/min) as mobile phase and ultraviolet detection at 236 nm. Acetonitrile was used to simultaneously deproteinize rat plasma and extract metformin. The assay was linear in the concentration range of 0.33 μg-16.6 μg/ml with co-efficient of correlation 0.994. The retention time was 4.7 min. The method was found to be precise (% CV < 15%), accurate and suitable for pharmacokinetic study of orally administered metformin in rats.

Metformin is a widely used oral antidiabetic agent and chemically it is 1,1-dimethyl biguanide (fig. 1)^1^. As its hypoglycemic effect depends upon its blood level, attempts have been made earlier to develop a precise method to estimate its concentration in biological matrix. These methods mostly employ ultraviolet or mass spectrometric detectors coupled to high-performance liquid chromatography (HPLC) using silica, cyano, cation-exchange and C_18_ columns 2–10. As metformin is a highly polar compound, previous investigators either used ion-pair solid phase extraction method or acidified the plasma before its extraction by an organic solvent or used capillary electrophoresis technique for its chromatographic separation^11^,^12^. Most of these reported methods are precise and accurate, however employ such specific columns or techniques which are not normally available in common laboratories. In view of this, a simple reverse phase HPLC method has been developed and validated as per USFDA guidelines^13^ to accurately and precisely estimate metformin in rat plasma using commonly available C_18_ column, ultraviolet detector, and acetonitrile for fundamental processes such as deproteinization of plasma and extraction of metformin.

**Fig. 1:**
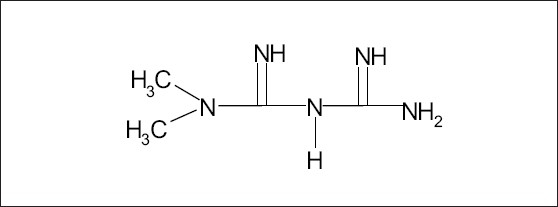
Structure of metformin.

## MATERIALS AND METHODS

Metformin was a generous gift by Zim Laboratories Ltd., Nagpur, India. Acetonitrile (HPLC grade) and ammonium acetate (AR grade), were purchased from Ranbaxy Chemicals, India (Rankem). Freshly double distilled, deionised water filtered through 0.45 μ nylon (47 mm, Pall Life Sciences, Mumbai, India) filter in Millipore unit (USA) was used throughout the experiments. Standard solution of metformin (1 mg/ml) was prepared in mobile phase and kept at 4-8°. Fresh stock solutions were prepared every week. The serial dilutions of stock solution were made to obtain working standard solutions of 0.5, 1.0, 2.0, 5.0, 10.0, 15.0, 20.0, 50.0 μg/ml strength. These solutions were freshly prepared every week and stored at 4-8°.

### Deproteinization of plasma and analyte extraction:

Sprague-Dawley rats (220-250 g) of either sex housed under the controlled conditions of temperature and humidity (25±2°, 55±2%) with dark/light (12/12 h) cycle and received a standard pelleted diet (Goldmohar brand, Lipton India Ltd.) and water *ad libitum*. The Institutional Animal Ethics Committee (IAEC) approved the use of animals. Whenever required, blood (1.0 ml) was withdrawn by micro-capillary technique from retro-orbital plexus^14^ under light ether anesthesia. It was collected in heparinized eppendorf tubes and centrifuged at 3000 × g to separate plasma. In order to deproteinize plasma, and subsequently extract metformin acetonitrile (1.0 ml) was added to plasma (0.5 ml) and the mixture was vortex-mixed for 30 s. After standing for 10 min, the mixture was centrifuged at 5000 × g for 10 min. The upper layer (about 100 μl) was separated and filtered through nylon membrane filters (0.22 μ, 13 mm). About 20 μl of filtrate was used for estimating metformin by HPLC method.

### Chromatography:

 The chromatographic study was carried out using Shimadzu 10AT/Vp HPLC system, having in-built degasser unit (DGU 14A), mixer unit (FCV 10AL) attached to solvent delivery module with low-pressure gradient pump (LC 10AT), Rheodyne injector port (2E, 7725i, 20μl loop) and UV/Vis detector (SPD 10A). The mobile phase was prepared by mixing 0.15 M ammonium acetate (pH 5.5) and acetonitrile in the ratio of 90:10; it was filtered through 0.45 μ membrane filter. The elution was carried out on C_18_ column (300 mm × 2.4 mm, Microbondapack) at a rate of 1 ml/min. Detection of metformin was carried out at 236 nm at ambient temperature. The data were interpreted using CSW (chromatographic work station) data acquisition software.

### Linearity and limit of quantitation:

 Calibration curve was established by spiking rat plasma (500 μl) with known amount of metformin to obtain 0.33, 1.66, 3.33, 5.0, 6.66, 16.60 μg/ml. The lowest concentration of the analyte that gives at least 5 times the response as compared with blank was considered as the limit of quantitation (LOQ). Quantitation of metformin in rat plasma was done by reading the analyte response against the calibration curve.

### Accuracy and precision:

In order to determine the intra-day and inter-day accuracy and precision, the concentration of metformin present in five replicates of plasma spiked with 3.33, 5.00, 6.66 μg/ml metformin was estimated by HPLC within a day or on three consecutive days. 85-115% accuracy and coefficient of variation values < 15% except at LLOQ (accuracy 80-120% and CV ≤20%) were considered acceptable.

### Recovery:

Recovery of metformin from plasma was estimated at 3.33, 5.00, 6.66 μg/ml concentrations by comparing peak area of spiked plasma standards with those of corresponding plain standards containing the corresponding concentration in mobile phase that represent 100% recovery.

### Stability:

Analyte stability in plasma was tested at 3.33, 5.00, 6.66 μg/ml concentrations in replicates of three for one freeze-thaw cycle, long term (30 days), and short term (24 h) stabilities. Initially, a plasma standard containing above concentrations were prepared and divided into four fractions. One fraction was analyzed immediately and its area was noted. The other three fractions were stored, two at -20°, and remaining at room temperature. The sample kept at room temperature was analyzed after 24 h. One of the samples kept at -20° was analyzed after one freeze-thaw cycle and the other was analyzed after one month storage. The peak area at the end of the study was compared with that of the standard and stability was calculated.

### Pharmacokinetic study:

For studying the pharmacokinetics of metformin by using the above developed method, metformin (320 mg/kg) was administered orally^15^ and the blood was withdrawn at 0.5, 1, 1.5, 2, 4, 8, 12 h time intervals. The study was carried out by sparse sampling design using 3 rats for each time point. Plasma obtained from each rat was stored at -20° until analyzed.

## RESULTS AND DISCUSSION

In the present method, acetonitrile was used to deproteinize plasma and simultaneously extract metformin. The recovery studies (Table 1) indicates that this solvent was effective to extract metformin from the spiked plasma. In the earlier attempts the investigators deproteinized the plasma by adding acid and subsequently extracted the analyte by using acetonitrile^6^. In fact, acetonitrile is a polar solvent and the present studies indicated that it has effectively deproteinized the plasma and hence, there is no necessity of separately using acid for this purpose. Since acetonitrile could also extract metformin, the present method appears comparatively simple for plasma preparation process.

**TABLE 1:** ACCURACY AND PRECISION OF THE METHOD FOR ESTIMATING METFORMIN CONCENTRATION IN PLASMA

Known concentration (μg/ml)	Concentration found (Mean ± S.D.; μg/ml)	Accuracy (%)	Precision (% CV)
Intra-day (n = 5)			
3.33	2.83±0.11	85.00	4.58
5.00	4.99±0.54	99.80	2.99
6.66	6.62±0.65	99.40	4.20
Inter-day (n =15)			
3.33	3.32 ±0.09	99.88	6.89
5.00	5.35±0.11	107.08	3.33
6.66	7.23±0.103	108.61	3.50

Each value is mean of 5 replicates; SD - Standard deviation; CV - Coefficient of variation

When metformin extracted by acetonitrile from plasma was injected onto C_18_ column using 0.15 M ammonium acetate (pH 5.5) and acetonitrile in the ratio of 90:10 as mobile phase, hled to a distinct curve for metformin at 4.7 min with good linearity indicating the preciseness and accuracy of the developed method. Most of the methods used potassium dihydrogen phosphate and acetonitrile in the mobile phase and used cyano or C_8_ column. However, our preliminary attempts revealed that use of ammonium acetate instead of potassium hydrogen phosphate in the mobile phase gives better resolution of metformin. This may be because metformin is a biguanide and possess amino groups and hence amino group possessing component in the mobile phase may be responsible for giving better resolution.

It was further noted that concentration of acetonitrile in mobile phase played a major role in the resolution of peak. At 10% concentration, the peak for metformin was optimally resolved with asymmetry of 0.9-1.4 and theoretical plates 18000-22000 tp/m. At higher concentration (>20%), the peak was broad whereas at lower concentration (< 10%), there was a tailing of peak which could be reduced by changing the pH of ammonium acetate buffer to 2.5. However, such acidic pH is not suitable for the life of the column. Therefore, 10% acetonitrile in the mobile phase was found more appropriate. In another experiment, methanol was used instead of acetonitrile for plasma preparation as well as in mobile phase. However, the ultimate peak height for metformin was very small and hence methanol was considered unsuitable.

**Fig. 2:**
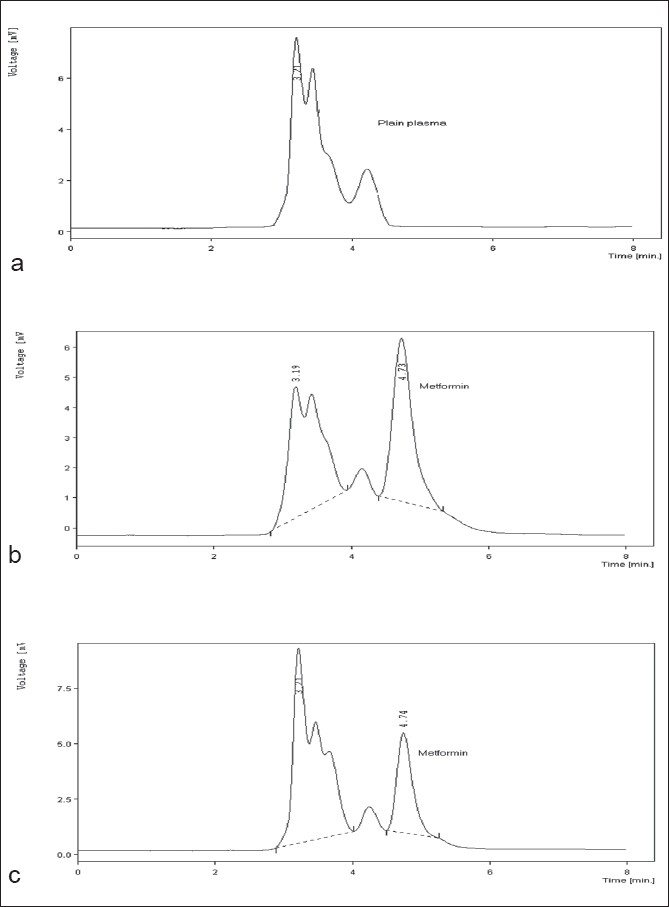
Chromatograms of metformin in rat plasma. a) Chromatogram of drug-free plasma, b) chromatogram of plasma spiked with metformin (5 μg/ml), c) chromatogram of metformin in rat plasma sample 30 min after oral administration of 320 mg/kg of metformin.

The typical chromatograms (fig. 2) obtained with the above-mentioned settings for drug-free plasma and plasma spiked with metformin indicates a distinct separation of the peak for metformin from plasma constituents. The retention time for metformin was found to be 4.7 min. Similar chromatogram was obtained for metformin extracted from the plasma, 30 min after the oral administration of 320 mg of metformin to the rats. The absence of any additional peak in this chromatogram indicates that either there is an absence of any metabolite in the extract or if any, it overlapped with the peak of plasma, possibly due to higher polarity and in no way affected the integrity of the peak for metformin.

For validation purpose, a concentration range of 0.33 μg/ml to 16.60 μg/ml was selected, as the plasma-level of 6-12 μg/ml of metformin was expected after an oral dose of 320 mg/kg to rats. A standard calibration curve was constructed by using linear regression analysis and it was found to be linear over the employed concentration range. The lower limit of quantitation was found to be 0.33 μg/ml at which percent co-efficient of variation of five replicates was 6.2. The linear regression equation was y = 22.46×-7.15 (r^2^ = 0.994). The inter-day and intra-day estimations of metformin revealed the reproducibility of the results irrespective of time and day. The calculated values of accuracy and precision (Table 1), recovery (Table 2) and stability (Table 3) of the method were well in accordance with the USFDA guideline for bioanalytical method validation^13^.

**TABLE 2:** ABSOLUTE RECOVERY OF METFORMIN FROM THE PLASMA SAMPLES

Concentration of metformin (μg/ml) (n=5)	Absolute Recovery % (Mean ± SD)	% CV
3.33	99.1±5.3	5.3
5.00	106.1±3.6	3.4
6.66	94.33±3.1	3.2

**TABLE 3:** STABILITY OF METFORMIN IN RAT PLASMA

Stability (n=3)	Concentration (mean±SD; μg/ml)		
	
	3.33	5.00	6.66
Freeze-thaw stability			
Initial	3.41±0.2	5.10±0.3	6.81±0.2
Final	3.28±0.3	5.00±0.4	6.52±0.1
Deviation (%)	-3.80	-1.96	-4.41
Short-term stability			
Initial	3.41±0.2	5.10±0.3	6.81±0.2
Final	3.32±0.1	4.89±0.4	6.78±0.4
Deviation (%)	-2.63	-4.11	-0.44
Long term stability			
Initial	3.41±0.2	5.10±0.3	6.81±0.2
Final	3.12±0.4	5.52±0.4	7.02±0.2
Deviation (%)	-8.50	+8.25	+3.08

**Fig. 3:**
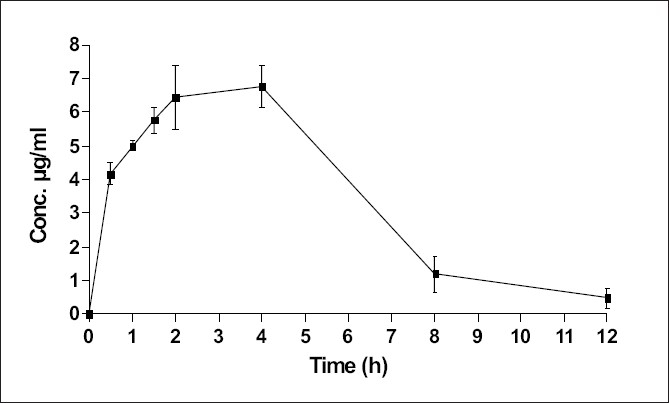
Plasma level-time profile of metformin. Metformin was administered at a dose of 320 mg/kg, p.o. in rats. Results are expressed as mean±SEM (n=3/time point).

The plasma level-time study of metformin in rats indicated highest metformin concentration in plasma to be 7.2±0.09 μg/ml (C_max_ ) at 2.5±0.115 h (T_max_ ) and AUC as 46.52±0.21 μg.h.ml^-1^ (fig. 3). Secondary pharmacokinetic parameters calculated are shown in Table 4.

**TABLE 4:** PHARMACOKINETIC PARAMETERS OF METFORMIN IN MALE RATS

Parameters	Mean±SD (n=3)
T_max_ (h)	2.50±0.12
C_max_ (μg/ml)	7.20±0.09
AUC_0-∞_ (μg.h.ml^-1^)	46.52±0.21
K_a_ (h^-1^)	0.30±0.057
T_1/2_ (h)	2.30±0.06
